# From glucose to histone modification: sex-specific metabolic responses to ketogenic therapy in VM/Dk mice

**DOI:** 10.3389/fnut.2025.1554743

**Published:** 2025-06-19

**Authors:** Sara E. Moss, Angela M. Poff, Apryl Moss, Janine M. DeBlasi, Dominic P. D’Agostino

**Affiliations:** ^1^Morsani College of Medicine, USF Health, Tampa, FL, United States; ^2^Direct Care and Wellness Treatment, Brooksville, FL, United States

**Keywords:** ketone metabolic therapy, exogenous ketones, epigenetic, metabolic therapy, insulin

## Abstract

**Introduction:**

The assessment of sex-specific effects in pre-clinical models is critical for improving the translatability of findings to clinical applications. However, preclinical studies often combine sexes or focus exclusively on one sex, including research utilizing syngeneic cancer models. Considering sex differences is particularly important in metabolic studies, as males and females exhibit distinct body compositions, hormone profiles, and metabolic demands.

**Methods:**

This study evaluates sex-specific metabolic responses to ketogenic therapies in VM/Dk mice, including assessments of glucose metabolism, ketone metabolism, and histone modifications linked to metabolism.

**Results:**

Our findings reveal significant sex differences in body weight, circulating metabolites, blood insulin levels, and histone modification profiles.

**Discussion:**

We demonstrate that male and female VM/Dk mice respond differently to ketogenic therapies, with responses varying based on the specific type of therapy applied.

## 1 Introduction

As the scientific and medical communities advance in the fight against cancer, novel therapies are regularly introduced. However, the literature usually reports the efficacy and side effects based on the overall drug study population without differentiating by sex, even though females and males are known to respond differently to treatments in the clinical environment. This combined population perspective fails to account for the fact that males and females often present with different disease phenotypes and assumes uniform sex responses to treatment. For example, zolpidem was originally FDA approved in 1992 with a single 10 mg dose approved for both men and women. In 2013, after several different studies on zolpidem blood levels 4 h post administration, immediate release dosing was reduced to 5 mg for women making it the first drug class with sex-specific dosage recommendations (1). Later studies have explored the differences of CYP3A4 metabolism on sex differences specifically looking at the pharmacokinetics and pharmacodynamics of zolpidem. In a 2021 study by Yoon et al. they noted higher zolpidem plasma concentrations across all time points studies for women vs. men (2).

Historically, there has been a systematic exclusion of sex as a biologic variable in clinical studies, and to address this, in 1993 the National Institutes of Health (NIH) and Congress instituted the NIH Revitalization Act (3). The NIH Revitalization Act mandated that females be included in NIH-funded clinical research. However, this did not address inclusion of males and female into preclinical research (3). In 2015, the NIH released a brief entitled, “Enhancing Reproducibility through Rigor and Transparency,” that stated rigorous experimental design should include the biological variables of sex, age, weight and underlying health conditions and to not include this information can affect the reproducibility of the experiment (NOT-OD-15-103). Furthermore, the NIH specifically states that “sex as a biological variable will be factored into research designs, analyses, and reporting in vertebrate animal and human studies” (NOT-OD-15-103). In 2017, 1 year after the policy was implemented, only 68% of NIH grantees surveyed sex as a biological variable that was important in experimental design and only 58% believe it improves rigor and reproducibility (3).

This combined population perspective is mirrored in many pre-clinical models of cancer research, such as the Viable Mottled/ Dunn and Kendall (VM/Dk) syngeneic cancer models. The VM/Dk mouse model originally bred from the Moredon Institute stock was characterized to have a spontaneously occurring astrocytoma, and has since been used to generate cell lines for various syngeneic cancer models (4–6). However, there are limitations to the published data sets from this model as most studies have used only females, only males, or did not report the sex distributions of mice used in the study (5–15). Pre-clinical studies on dietary therapies in VM/Dk cancer models have also commonly utilized only one sex or did not report sex distributions in the treatment groups (12–15). One study has accounted for potential sex effects of ketone supplementation in a VM/Dk model of cancer cachexia and noted a discrepancy between female and male adipose tissue wasting (16). Even with this study, sex specific responses to ketogenic therapies in the VM/Dk model were not assessed.

Sex dimorphisms in body composition and dietary responses have been reported in humans, with males and females having different body compositions with similar diet compositions, and an indication that males may have higher capacity for carbohydrate metabolism while females have higher capacity for lipid metabolism (17). Sex plays an important role in the physiological and therapeutic effect of ketogenic therapies. Males and females have hormonal differences that can affect metabolic pathways, ketogenesis, lipid metabolism, and mitochondrial function. For example, it’s been reported that following meal consumption, females exhibited higher levels of ketone bodies compared to males in both healthy, normolipidemic patients and untreated hyperlipidemic patients (18). Another study also observed that female C57BL/6 J mice on a ketogenic diet had significantly higher circulating beta-hydroxybutyrate (BHB) levels compared to male mice (19). This suggests that there can be sex-specific variations in ketone production and utilization, which can have implications in understanding metabolic disorders and development of dietary intervention guidelines. These differences can be relevant in cancer treatment, as elevated ketone levels could enhance metabolic stress on tumors and influence therapeutic outcomes by targeting the Warburg effect and associated metabolic and redox signaling (15, 16, 20).

Moreover, sex hormones like estrogen and progesterone can also modulate the effects of ketogenic therapy. Estrogen has been reported to affect mitochondrial activity and oxidative stress (21), both of which can influence response to metabolic therapies. These hormonal effects can also interact with the immune system, affecting inflammation and the microenvironment in sex-specific ways (22).

Finally, energy expenditure and body composition can also affect how males and females respond to ketogenic therapies. Females tend to have less lean mass and more fat stores compared to males, which can affect the production and use of ketones (23). The observed higher fat mass to lean mass ratio in females, even when matched for BMI, is due to differences in sex hormones and fat distribution patterns. The greater fat mass provides a larger reservoir of triglycerides, which can enhance hepatic ketogenesis during periods of carbohydrate restriction or fasting. In contrast, lower lean mass in females may reduce glucose disposal capacity and increase reliance on lipid-derived fuels such as ketone bodies for energy during metabolic stress. These factors can affect the sustainability of a ketogenic diet over time and warrants the need for personalized treatment approaches that account for the biological differences between males and females.

To better understand the effects of cancer treatments and metabolic therapies, an effort should be made to understand the sex differences in pre-clinical models. Metabolic and genetic variations can influence how a specific therapy is metabolized in both human and rodent subjects. The genetic and epigenetic mechanisms of sex differentiation affect key factors including growth, lifespan, immunity and metabolism (22). These factors collectively influence cancer progression, response to treatment, as well as overall survival (22).

In this study, we aim to establish a foundation for assessing sex-specific metabolic differences in the VM/Dk mouse model. Our goal was to assess sex differences in glucose metabolism, ketone metabolism, and histone modifications linked to metabolic pathways under three dietary conditions in the VM/Dk mouse model. Our findings provide insights that will shape future research on sex differences in preclinical cancer models.

## 2 Materials and methods

### 2.1 Animals

Breeding pairs of the VM/Dk inbred strain of mice were received as a gift from T. Seyfried of Boston College and were used to establish and propagate a mouse colony in the University of South Florida Morsani College of Medicine Vivarium according to standard husbandry protocol (climate controlled, 12 h light/dark cycle). Male and female VM/Dk mice between 15 and 19 weeks of age were age matched across groups, so that 5 males and 5 females were assigned to each diet. Tails were wounded under isoflurane 18 days before the start of the study to facilitate blood metabolite measurements, and the mice were handled 4 times a week for 2 weeks leading up to the start of treatment to acclimatize them to the procedures used. The tail wound was aggravated during metabolite measurements to measure blood lactate (Lactate Plus, Nova Biomedical), glucose (Precision Xtra, Abbott Laboratories), and R-BHB (Precision Xtra, Abbott Laboratories) levels. Blood metabolites were measured every 7 days 4 h after the start of the light cycle. Food was measured every day for the first 7 days and every other day for days 9–42 to ensure sufficient food was present for ad-libitum feeding. However, due to the mice scattering and burying the food, accurate calculations of diet consumption could not be made beyond ensuring ad-libitum access. Body weight was measured every day for the first 7 days, every other day for days 9–21, and once a week for days 28–42 to ensure the mice maintained a steady weight. At the end of the study, 4 h into light cycle, blood was collected via submandibular puncture for fed insulin measurements. Mice were humanely euthanized via CO₂ asphyxiation in accordance with Institutional Animal Care and Use Committee (IACUC)–approved protocols (Protocol #IS00009972) and in compliance with U.S. federal guidelines. The procedure followed the standard operating protocol of the University of South Florida Comparative Medicine Program (SOP #401.5). Briefly, animals were placed in a clean, dry chamber with a tight-fitting lid. Pure CO₂ was introduced at a flow rate of 4 L/min, displacing approximately 30–70% of the chamber volume per minute to minimize distress. After confirmation of respiratory and cardiac arrest, euthanasia was completed with cervical dislocation to ensure death, and immediately perfused with 10x PBS.

Tissues were then harvested, weighed, and flash frozen in liquid nitrogen.

### 2.2 Diet formulations

Standard Diet (SD): Powdered standard rodent chow (Teklad Global 18% protein rodent diet; Wisconsin, US) was mixed with water at a 1 g:1 mL ratio to form a smooth paste (Table 1).

**Table 1:** Diet formulations.

Diet	Standard diet (Teklad 2018)	Ketogenic diet (Teklad KD2)	15%BHB:MCT supplemented standard diet
Protein (%kcal)	24	22.4	5.6
Carbohydrate (%kcal)	58	0.5	36.6
Fat (%kcal)	18	77.1	34.3
Fat source	Soybean oil	Medium-Chain-Triglycerides (MCT) oil, flaxseed oil, canola oil	Soybean oil, fiber bound MCTs
Na-BHB (%kcal)	0	0	22.8

Standard diet mice received Teklad Global 18% Protein Rodent Diet (Teklad 2018) which has a %kcal fat:protein:carbohydrate composition of 18:24:58. Teklad 2018 was mixed with water at a 1 g:1 mL ratio to match the paste-like consistency of the ketogenic diet. Ketogenic diet assigned mice received Ketogenic Diet 2 (MCT, Flax, Canola)-TD.10911 (Teklad KD2) which has a %kcal fat:protein:carbohydrate composition of 77.1:22.4:0.5. 15%BHB:MCT supplemented standard diet assigned mice received Teklad 2018 supplemented with Na-BHB and fiber bound MCTs so that for every 35 g of Teklad 2018 there were 7.5 g Na-BHB and 7.5 g MCTs. Teklad 2018 + Na-BHB + MCT was mixed with water at a 1 g:1 mL ratio to match the paste-like consistency of the ketogenic diet, creating the 15%BHB:MCT diet.

Ketogenic Diet (KD): Ketogenic Diet (high in medium chain triglyceride oil (MCT), Flax oil)-TD.10911 (Teklad KD2; Wisconsin, US).

15% BHB:MCT: Powdered standard rodent chow was mixed with powdered MCT (ABITEC Nutrispearse-MCT 70; Ohio, US), Na-BHB (racemic) (NNB Nutrition; California, United States), and water at an 8.5 g chow:0.75 g MCT:0.75 g BHB:10 mL water ratio.

### 2.3 Fed state insulin ELISA

Blood collected before euthanasia (4 h into light cycle) remained at room temperature for 10 min before being spun down at 2,000 g for 10 min in a centrifuge cooled to 4°C. The serum was then extracted via micropipette, placed in a separate tube, and flash frozen in liquid nitrogen. Later, the serum was thawed to room temperature and used in the Rat/Mouse Insulin ELISA Kit (Millipore Sigma, EZRMI-13K) using the included plasma matrix buffer as a precaution.

### 2.4 Histone extractions and dot blot assays

Histones were extracted from flash frozen hippocampus and bicep samples collected at end of life using the EpiQuik Total Histone Extraction Kit (EpiGenTek OP-0006-100). Using pre-cut nitrocellulose paper marked with graphite pencil, 2uL of each extract were loaded in duplicate for each modification assessed. Bovine-Serum Albumin (BSA) was diluted in water to create a known protein concentration curve for each blot. The blots were allowed to dry for at least 2 h before they were stained with S-Ponceau. The blots were then washed with water for 2 min to remove excess S-Ponceau, placed in protective plastic, and scanned in a – printer. These images were imported to Image-J software and used to calculate total protein loaded in each sample, using the BSA dilutions as the standard curve for the S-Ponceau signal. The remaining S-Ponceau was washed away, then the blots were blocked overnight at 4°C with 5% NFDM:TBST under agitation. All antibodies were diluted in 5% NFDM:TBST: Pan-Kac (PTM bio PTM-105) 1:2000; H3K4Me3 (PTM bio PTM-613) 1:2000; H3K9me3 (PTM bio PTM-616) 1:2000. The blots were incubated in the primary antibodies overnight at 4°C under agitation. The blots were then washed in TBST 3 times for 10–15 min each wash, then incubated in secondary for 1 h. The blots were then washed 5 times in TBST for 10–15 min each. Using standard ECL (Thermosci) methods, the blots were imaged using the ChemiDoc XRS system. These images were then imported into Image-J software and the signal intensity of each sample was recorded as integrated density. The signal intensity was then divided by the total loaded protein (via the S-Ponceau signal) as a normalization.

### 2.5 Statistics

All statistics were performed using SPSS 29 software as indicated in the results section. Briefly, effects of time, diet, and sex were assessed through repeated measures 2-way ANOVAs, followed by pairwise comparisons for simple effects within groups. For single timepoint measurements (insulin and histone modifications), effects of sex and diet were assessed using univariate 2-way ANOVAs followed by pairwise comparisons for simple effects within groups. *p* < 0.05 was used to determine significance. An omnibus ANOVA was used to assess overall group-level differences across factors before conducting pairwise comparisons, ensuring that any reported simple effects were grounded in a statistically significant overall model. Graphs were made using GraphPad Prism.

## 3 Results

### 3.1 Effects of sex and diet on bodyweight

**Figure 1:**
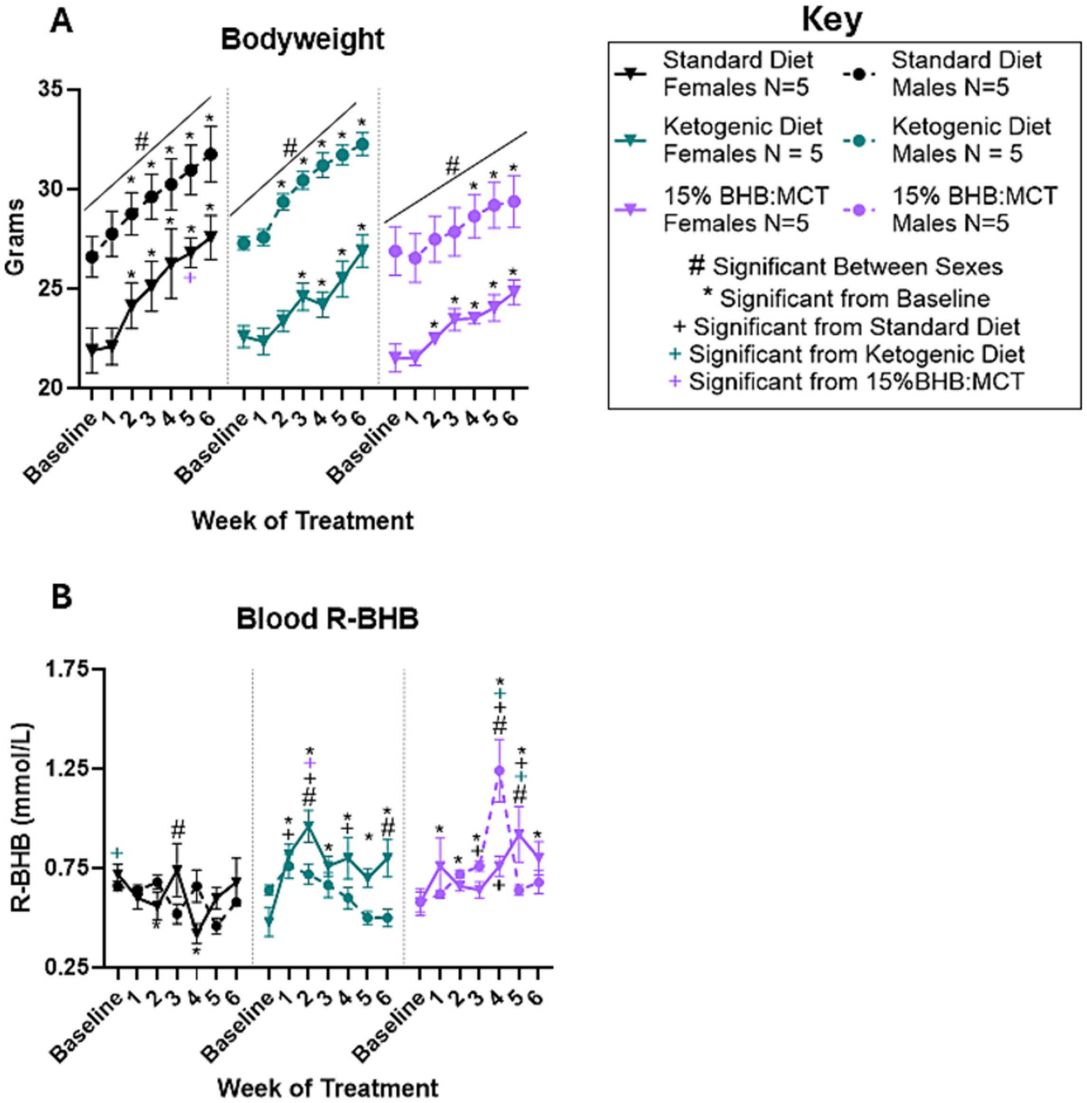
Female and male VM/Dk bodyweight and R-BHB response to 6-week diet administration. *N* = 5 for all groups as indicated by key, *p* < 0.05 used to determine significance, and error bars are SEM. **(A)** Results of repeated measures 2-way ANOVA show that females of all groups weighed less than their male counterparts at every timepoint (indicated with black # over line). All mice gained weight over time compared to baseline. Standard diet (SD) males, ketogenic diet (KD) males, SD females, and 15%BHB:MCT (BM) females, weighed more at weeks 2–6 than at baseline. KD females weighed more at weeks 3–6 and BM males weighed more at weeks 4–6 compared to baseline (indicated by *). SD females weighed more than BM females at week 5. **(B)** Results of repeated measures 2-way ANOVA show that SD females had higher R-BHB than KD females at baseline, lower R-BHB at week 3 than SD males, and lower R-BHB at weeks 2 and 4 compared to baseline. KD females had higher R-BHB than KD males at weeks 2 and 6, higher R-BHB than SD females at weeks 1, 2, and 4, and higher R-BHB than BM females at week 2. KD females had higher R-BHB levels at weeks 1, 2, 3, 4, 5, and 6 compared to baseline. KD males showed no difference compared SD males at any timepoint. SD males and KD males showed no change in R-BHB compared to baseline. It is important to note that ketogenic diet males only have 4 measurements for the week 3 timepoint due to instrument error. BM females had lower R-BHB than BM males at week 4, higher R-BHB than BM males at week 5, higher R-BHB than SD females at weeks 4 and 5, and higher R-BHB than KD mice at week 5. BM females had higher R-BHB at weeks 1, 5, and 6 compared to baseline. BM males had higher R-BHB than SD males at week 3 and 4, higher R-BHB compared to KD male mice at week 4, and higher R-BHB at weeks 2, 3, and 4 compared to baseline.

Bodyweight was measured at baseline and throughout the study as described in Section 2 (Figure 1A). Results of repeated measures 2 way ANOVA indicate an interaction between week of treatment and diet assignment [*F*(8.176, 98.109) = 2.995, *p* < 0.05], but no interaction between week of treatment and sex [*F*(4.088, 98.109) = 0.616, *p* > 0.05] or between week of treatment, diet assignment, and sex [*F*(8.176, 98.109) = 1.423, *p* > 0.05]. Assessment of between subject effects indicate an effect of sex on bodyweight [*F*(1, 24) = 52.523, *p* < 0.05], but no effect of diet assignment [*F*(2, 24) = 2.255, *p* > 0.05] or interactive effects of sex and diet assignment [*F*(2, 24) = 0.260, *p* > 0.05]. Pairwise comparisons show that female VM/Dk mice had lower bodyweight compared to male VM/Dk mice at every timepoint in all three groups (*p* < 0.05). At week 5, 15% BHB:MCT assigned female mice had a lower bodyweight than SD female mice (*p* < 0.05). SD female and male mice had higher bodyweights at weeks 2,3, 4, 5, and 6 compared to baseline (*p* < 0.05). KD female mice had higher bodyweights at weeks 3, 4, 5, and 6 compared to baseline (*p* < 0.05), and KD male mice had higher bodyweights at weeks 2, 3, 4, 5, and 6 compared to baseline (*p* < 0.05). 15% BHB:MCT diet female mice had higher bodyweights at weeks 2, 3, 4, 5, and 6 compared to baseline (*p* < 0.05), and 15%BHB:MCT male mice had higher bodyweights at weeks 4, 5, and 6 compared to baseline (*p* < 0.05).

### 3.2 Effects of sex and diet on circulating R-BHB

Blood R-BHB was measured at baseline and throughout the study as described in Section 2 (Figure 1B). Results of repeated measures 2 way ANOVA indicate an interactive effect between week of treatment, diet assignment, and sex [*F*(12, 138) = 3.069, *p* < 0.05] on circulating R-BHB levels. Assessment of between subject effects indicate an effect of diet assignment on circulating R-BHB levels [*F*(2, 23) = 9.603, *p* < 0.05], but no effect of sex [*F*(1, 23) = 3.240, *p* > 0.05] or interactive effects of sex and diet assignment [*F*(2, 23) = 3.276, *p* > 0.05]. Pairwise comparisons show that SD female VM/Dk mice had higher circulating R-BHB levels than male SD mice at week 3 (*p* < 0.05). KD female mice had higher circulating R-BHB levels at weeks 2 and 6 (*p* < 0.05) compared to male KD mice. 15%BHB:MCT female VM/Dk mice had lower circulating R-BHB levels at week 4 (*p* < 0.05) and higher circulating R-BHB levels at week 5 (*p* < 0.05) compared to male 15%BHB:MCT mice. At baseline, SD assigned female mice had higher circulating R-BHB levels compared to KD female mice (*p* < 0.05). Female KD mice had higher circulating R-BHB levels compared to SD female mice at weeks 1, 2, and 4 (*p* < 0.05). Female KD mice had higher circulating R-BHB levels compared to 15%BHB:MCT female mice at week 2 (*p* < 0.05), and lower circulating R-BHB levels compared to 15%BHB:MCT female mice at week 5 (*p* < 0.05). Female 15%BHB:MCT mice had higher circulating R-BHB levels compared to SD female mice at weeks 4 and 5 (*p* < 0.05). 15%BHB:MCT male mice had higher circulating R-BHB levels compared to SD at week 3 (*p* < 0.05) and compared to both SD and KD male mice at week 4 (*p* < 0.05). Male KD mice showed no difference compared to male SD mice at any timepoint (*p* > 0.05). SD female mice had lower circulating R-BHB levels at weeks 2 and 4 compared to baseline (*p* < 0.05). KD female mice had higher circulating R-BHB levels at weeks 1, 2, 3, 4, 5, and 6 compared to baseline (*p* < 0.05). 15%BHB:MCT female mice had higher circulating R-BHB levels at weeks 1, 5, and 6 compared to baseline (*p* < 0.05). 15%BHB:MCT male mice had higher circulating R-BHB levels at weeks 2, 3, and 4 compared to baseline (*p* < 0.05). Standard diet males and KD males showed no change in R-BHB compared to baseline (*p* > 0.05). It is important to note that KD males only have 4 measurements for the week 3 timepoint due to instrument error.

### 3.3 Effects of sex and diet on circulating glucose

**Figure 2:**
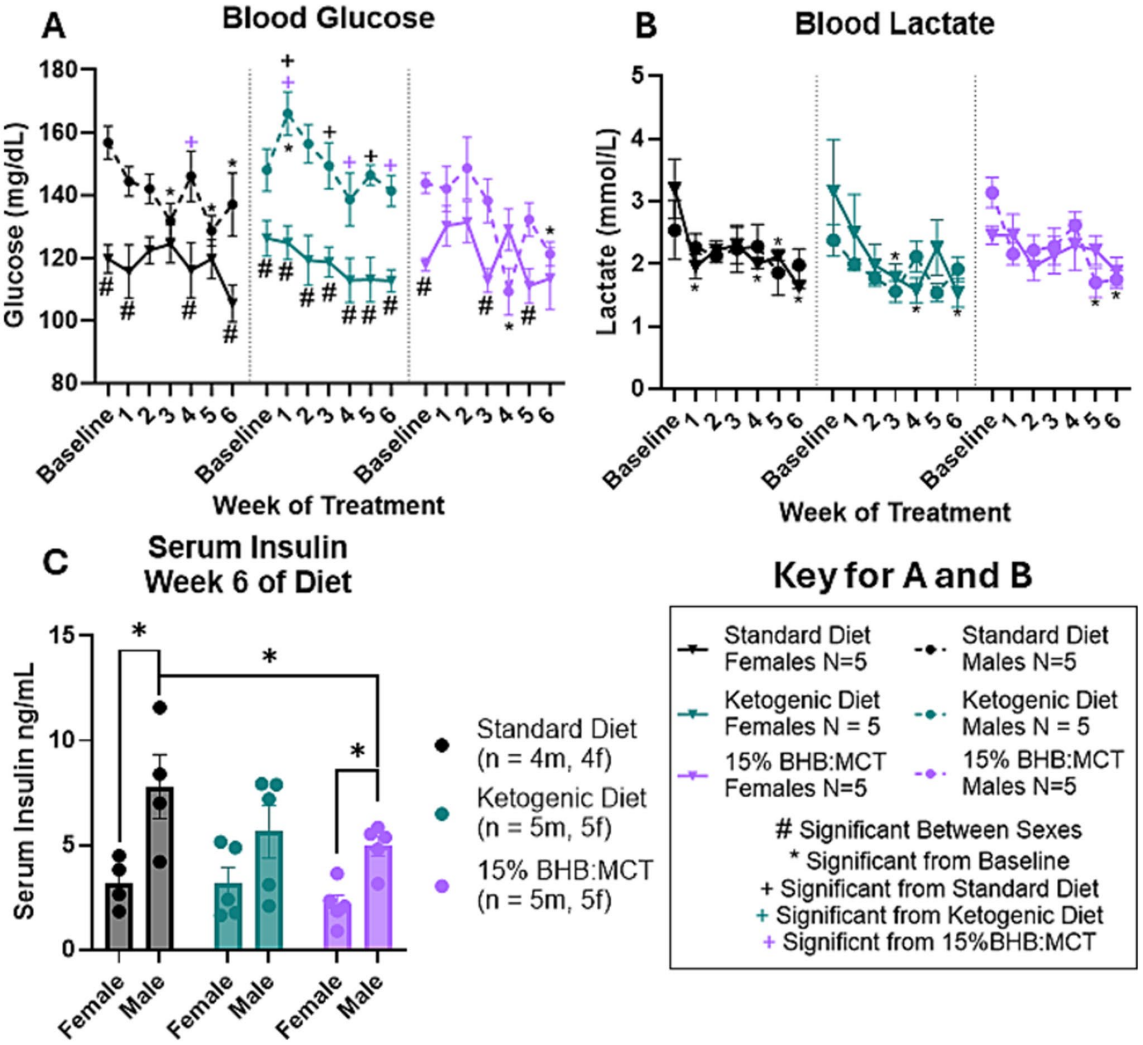
Female and male VM/Dk glucose, lactate, and insulin response to 6-week diet administration. *N* = 5 for all groups except standard diet (SD) groups in C (*N* = 4) as indicated by keys, *p* < 0.05 used to determine significance, and error bars are SEM. **(A)** Results of repeated measures 2-way ANOVA show SD females had lower glucose than SD males at baseline and weeks 1, 4, and 6. Ketogenic diet (KD) females had lower glucose than KD males at all timepoints. 15%BHB:MCT (BM) female mice had lower glucose than BM males at baseline and weeks 3 and 5. Female mice showed no significant difference in circulating glucose across diets or time. SD males had higher glucose than BM males at week 4, and lower glucose at weeks 3, 5, and 6 compared to baseline. KD males had higher glucose than SD males at weeks 1, 3, and 5, higher glucose than BM males at weeks 1, 4, and 6, and had higher glucose at week 1 compared to baseline. BM males had lower glucose at weeks 4 and 6 compared to baseline. **(B)** Results of repeated measures 2-way ANOVA show SD females had lower lactate at weeks 1, 4, 5, and 6 compared to baseline. KD females had lower lactate at weeks 3, 4, and 6 compared to baseline. BM males had lower lactate at weeks 5 and 6 compared to baseline. SD males, KD males, and BM females showed no change in lactate across weeks. It is important to note that BM males only have 2 lactate measurements for week 6 due to instrument error. **(C)** Results of univariate 2-way ANOVA show SD males have higher insulin than SD females, BM males have higher insulin than BM females, and BM males have lower insulin than SD males.

Blood glucose was measured at baseline and throughout the study as described in Section 2 (Figure 2A). Results of repeated measures 2 way ANOVA indicate an interactive effect between week of treatment, diet assignment, and sex [*F*(12, 144) = 2.241, *p* < 0.05]. Assessment of between subject effects indicate an interactive effect between sex and diet assignment [*F*(2, 24) = 5.267, *p* < 0.05] on circulating glucose levels. Pairwise comparisons show that SD female mice had lower circulating glucose levels compared to male mice at baseline, week 1, week 4, and week 6 (*p* < 0.05). KD female mice had lower circulating glucose levels compared to male VM/Dk mice at all timepoints (*p* < 0.05). 15%BHB:MCT female mice had lower circulating glucose levels compared to male mice at baseline, week 3, and week 5 (*p* < 0.05). Female mice showed no difference in circulating glucose levels across diets (*p* > 0.05). At weeks 1, 3, and 5, KD male mice had a higher circulating glucose than SD male mice (*p* < 0.05). At weeks 1, 4, and 6, KD male mice had a higher circulating glucose than 15%BHB:MCT male mice (*p* < 0.05). 15%BHB:MCT mice had lower circulating glucose than SD mice at week 4 (*p* < 0.05). Female mice showed no change across time compared to baseline in all diet groups (*p* > 0.05). SD male mice had lower circulating glucose at weeks 3, 5, and 6 compared to baseline (*p* < 0.05). KD male mice had higher circulating glucose at week 1 compared to baseline (*p* < 0.05). 15% BHB:MCT diet male mice had lower circulating glucose at weeks 4 and 6 compared to baseline (*p* < 0.05).

### 3.4 Effects of sex and diet on circulating lactate

Blood lactate was measured at baseline and throughout the study as described in Section 2 (Figure 2B). Results of repeated measures 2 way ANOVA indicate an effect of week of treatment on circulating lactate levels [*F*(3.552, 74.592) = 7.010, *p* < 0.05]. There is no indication of an interaction between week of treatment and diet assignment [*F*(7.104, 74.592) = 0.409, *p* > 0.05], between week of treatment and sex [*F*(3.552, 74.592) = 0.902, *p* > 0.05] or between week of treatment, diet assignment, and sex [*F*(7.104, 74.592) = 1.121, *p* > 0.05]. Assessment of between subject effects indicate no effect of sex [*F*(1, 21) = 0.004, *p* > 0.05], diet assignment [*F*(2, 21) = 1.887, *p* > 0.05], or interactive effects of sex and diet assignment [*F*(2, 21) = 0.933, *p* > 0.05]. Pairwise comparisons show that SD female mice have lower circulating lactate levels at weeks 1, 4, 5, and 6 compared to baseline (*p* < 0.05). KD female mice have lower circulating lactate levels at weeks 3, 4, and 6 compared to baseline (*p* < 0.05). 15%BHB:MCT male mice have lower circulating lactate levels at weeks 5 and 6 compared to baseline (*p* < 0.05). Male SD, male KD, and female 15%BHB:MCT mice showed no change in lactate across weeks (*p* > 0.05). It is important to note that 15%BHB:MCT males only have 2 lactate measurements for the week 6 timepoint due to instrument error.

### 3.5 Effects of sex and diet on circulating insulin

Serum insulin was measured at baseline and throughout the study as described in Section 2 (Figure 2C). Results of univariate 2-way ANOVA indicate an effect of sex on serum insulin levels [*F*(1, 22) = 19.452, *p* < 0.05], and no effect of diet assignment [*F*(2, 22) = 2.191, *p* > 0.05]. Assessment of simple effects across sexes shows that male SD mice have higher serum insulin levels than female SD mice (*p* < 0.05), and that males assigned 15%BHB:MCT also had higher insulin than their female counterparts (*p* < 0.05). Assessment of simple effects across diet assignments shows 15%BHB:MCT males have lower serum insulin levels than their SD counterparts (*p* < 0.05).

### 3.6 Effects of sex and diet on histones extracted from the hippocampus

**Figure 3:**
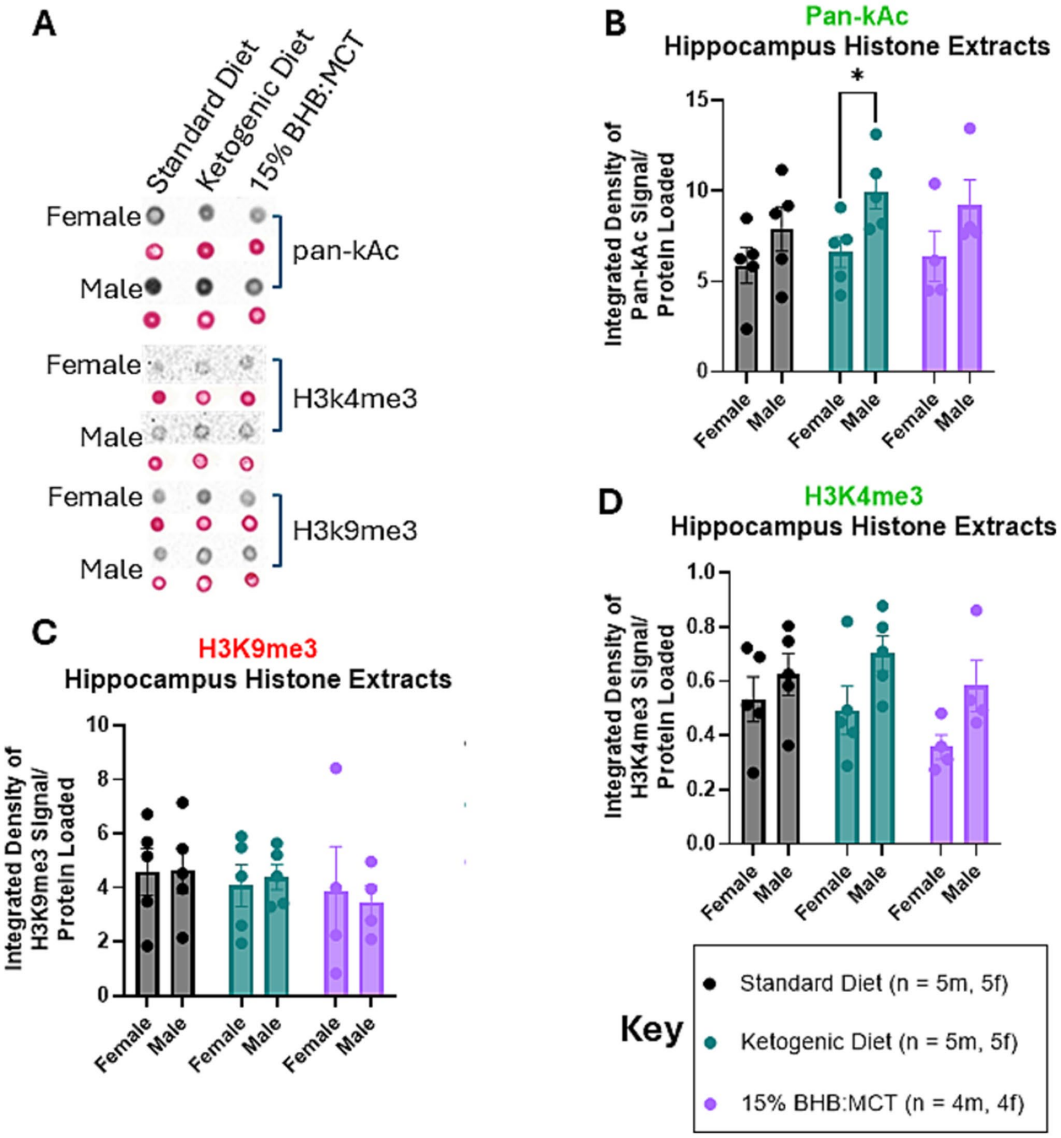
Female and male VM/Dk histone modifications in the hippocampus. *N* = 5 for all groups except 15% BHB:MCT (BM) groups (*N* = 4) as indicated by key, *p* < 0.05 used to determine significance, and error bars are SEM. **(A)** Representative images of dot blot analyses shown in **(B–D)**. Corresponding S-Ponceau shown below each signal representative. **(B)** Results of univariate 2-way ANOVA show that ketogenic diet (KD) had higher pan-Kac than KD females. **(C)** Results of univariate 2-way ANOVA show no difference in H3K4me3 levels between groups or within groups. It is important to note the omnibus ANOVA indicates an effect of sex on H3K4me3 [*F*(1, 22) = 7.490, *p* < 0.05]. **(D)** Results of univariate 2-way ANOVA show no difference in H3K9me3 levels between groups or within groups.

Histones were extracted from flash frozen hippocampus tissue and assessed for pan-Ac (Figure 3B), H3K4me3 (Figure 3D), and H3K9me3 (Figure 3C) as described in Section 2 (Figures 3A–D). Pan-Ac and H3K4me3 are commonly known marked for active chromatin, and H3K9me3 is commonly associated with inactive chromatin. Results of univariate 2-way ANOVA indicate an effect of sex on pan-Kac levels in histones extracted from the hippocampus [*F*(1, 22) = 8.681, *p* < 0.05], and no effect of diet assignment [*F*(2, 22) = 0.852, *p* > 0.05]. Assessment of simple effects showed that males assigned a KD have higher pan-Kac than their female counterparts (*p* < 0.05). Results of univariate 2-way ANOVA indicate an effect of sex on H3K4me3 levels in histones extracted from the hippocampus [*F*(1, 22) = 7.490, *p* < 0.05], and no effect of diet assignment [*F*(2, 22) = 1.430, *p* > 0.05]. Assessment of simple effects within diet groups show no difference in H3K4me3 levels across sexes (*p* > 0.05). Results of univariate 2-way ANOVA indicate no effect of sex on H3K9me3 levels in histones extracted from the hippocampus [*F*(1, 22) = 0, *p* > 0.05], and no effect of diet assignment [*F*(2, 22) = 0.537, *p* > 0.05].

### 3.7 Effects of sex and diet on histones extracted from the bicep

**Figure 4:**
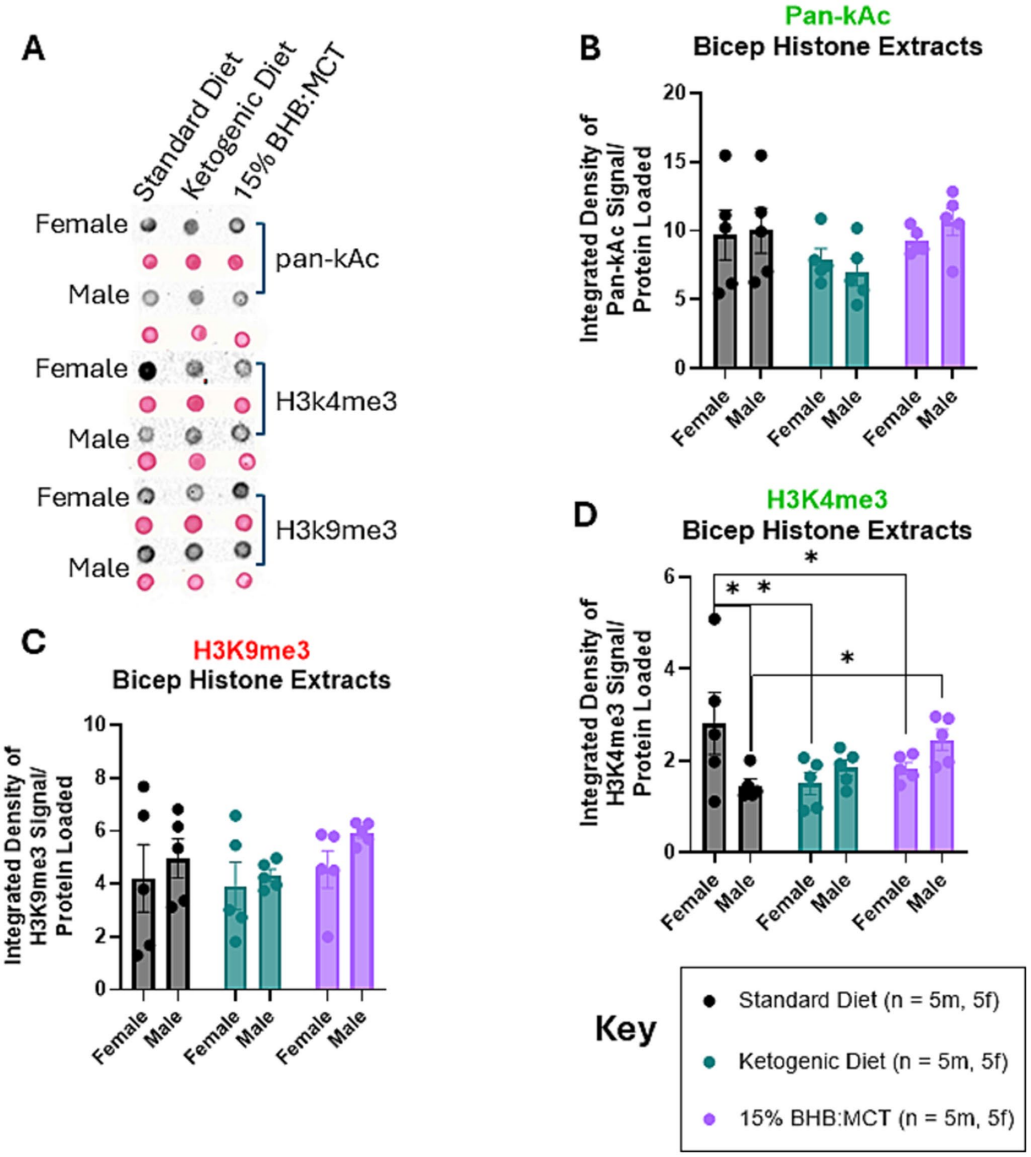
Female and male VM/Dk histone modifications in the bicep. *N* = 5 for all groups as indicated by key, *p* < 0.05 used to determine significance, and error bars are SEM. **(A)** Representative images of dot blot analyses shown in **(B–D)**. Corresponding S-Ponceau shown below each signal representative. **(B)** Results of univariate 2-way ANOVA show no difference in pan-kAc levels between groups or within groups. **(C)** Results of univariate 2-way ANOVA show standard diet (SD) females had higher H3K4me3 than both ketogenic diet (KD) and 15%BHB:MCT (BM) females, while SD males had lower H3K4me3 than BM males. SD females also had higher H3K4me3 than male standard diet mice. **(D)** Results of univariate 2-way ANOVA show no difference in H3K9me3 levels between groups or within groups.

Histones were extracted from flash frozen hippocampus tissue and assessed for pan-Ac (Figure 4B), H3K4me3 (Figure 4D), and H3K9me3 (Figure 4C) as described in Section 2 (Figures 4A–D). Results of univariate 2-way ANOVA indicate no effect of sex on pan-Kac levels in histones extracted from the bicep [*F*(1, 24) = 0.077, *p* > 0.05], and no effect of diet assignment [*F*(2, 24) = 2.732, *p* > 0.05]. Results of univariate 2-way ANOVA indicate an interactive effect of sex and diet assignment on H3K4me3 levels in histones extracted from the bicep [*F*(2, 24) = 5.399, *p* < 0.05]. Assessment of simple effects across diet groups show that SD females have higher H3K4me3 than both KD and 15%BHB:MCT females (*p* < 0.05), while male SD mice have lower H3K4me3 than 15%BHB:MCT males (*p* < 0.05). Female SD mice also have higher H3K4me3 than male SD mice (*p* < 0.05). Results of univariate 2-way ANOVA indicate no effect of sex on H3K9me3 levels in histones extracted from the bicep [*F*(1, 24) = 1.810, *p* > 0.05], and no effect of diet assignment [*F*(2, 24) = 1.049, *p* > 0.05].

## 4 Discussion and conclusion

Our study observed sex-specific differences in how VM/Dk mice respond to ketogenic therapies. Despite all groups eventually gaining weight over 6 weeks, at various time points the rate of weight change varied by sex and diet. Specifically, female mice on the ketogenic diet (KD) showed a delayed weight gain, taking an extra week to exceed baseline weight, whereas males on the 15%BHB:MCT-supplemented diet required two extra weeks to gain weight. Notably, 15%BHB:MCT females had a lower body weight than standard diet females by week 5. These patterns suggest that males and females differ in metabolic adaptation to carbohydrate restriction and perhaps exogenous ketone supplementation. This divergence may reflect underlying sex-specific differences in metabolic flexibility and energy utilization. Indeed, healthy females are reported to be more metabolically flexible – shifting between fat and glucose oxidation more efficiently – than males (24). On average, and across species, females have higher adipose mass and lower lean mass than males, which can influence ketone production and utilization. These inherent physiological differences could explain why a strict KD slowed weight gain predominantly in females (perhaps due to greater reliance on fat stores), whereas the ketone-supplemented diet impacted males more (potentially due to faster adaptation to carbohydrate restriction). In essence, each sex had a unique metabolic response to the diets, underscoring the importance of considering sex as a biological variable in metabolic interventions.

One of the most striking sex-specific findings was the consistently elevated R-*β*-hydroxybutyrate (R-BHB) levels in KD females compared to all other groups, including KD males. Female VM/Dk mice maintained higher ketone bodies throughout the study, a result that aligns with prior reports in other models. For example, female C57BL/6 J mice on a ketogenic diet were found to have significantly higher circulating BHB than males, and clinical observations have noted higher ketone levels in women under certain metabolic conditions. This suggests that female physiology may favor ketone body production or utilization. Higher ketone availability in females could have important implications. Ketone bodies can serve as alternative energy sources but also impose metabolic stress on tumors by limiting glucose and altering redox status. In cancer contexts, elevated ketones might enhance therapeutic outcomes by fueling normal tissues while starving glycolysis-dependent tumors (25). Thus, the female-biased ketone response observed here might translate to a greater inherent capacity to induce anti-tumor metabolic stress, although this remains to be fully tested in tumor-bearing VM/Dk models. In contrast, blood glucose levels showed an opposing trend, with females overall maintaining lower glucose than males across diets. Standard diet and 15%BHB:MCT males had higher glycemia than their female counterparts at multiple time points, whereas KD males exhibited significantly higher glucose than KD females at all time points. Notably, KD males failed to show the decline in glucose by week 6 that was seen in other groups. This sustained higher glycemia in KD males, despite carbohydrate restriction, suggests sex-specific regulation of gluconeogenesis, glycogenolysis, or peripheral insulin sensitivity under ketosis. We speculate that male mice on the KD had a greater counter-regulatory response (e.g., increased gluconeogenesis), resulting in higher blood glucose. By contrast, females (even on KD) maintained stable glucose, indicating tighter glycemic control, even with no overt changes in physical activity. These observations are consistent with reports that male rodents are more prone to what appears to be physiological insulin resistance when challenged with ketogenic or high-fat diets, whereas this response is attenuated in females (26). Our data similarly suggest that KD males may have experienced a degree of physiological insulin resistance or less metabolic flexibility (glycolytic flux) that prevented them from lowering blood glucose over time. In combination with the ketone findings, this highlights a fundamental sex difference in fuel utilization: females readily enter a ketosis, fat-oxidative state with lower glucose and higher ketones, whereas males on the same diet tend to maintain higher glucose and more moderate ketosis. It was not determined in our study if males have high ketone uptake/utilization. Such differences could stem from hormonal influences (estrogen vs. androgen) on metabolic pathways or from sex-specific enzyme expression, as discussed below.

Apart from ketone and glucose disparities, lactate levels did not significantly differ by sex or diet, indicating that glycolytic flux and anaerobic metabolism were similarly regulated in all groups. However, insulin levels showed a clear sex dimorphism. Male VM/Dk mice had higher circulating insulin than females, especially on the standard and 15%BHB:MCT diets. Females in these groups maintained lower insulin, consistent with their lower glycemic levels. Interestingly, 15%BHB:MCT-fed males had reduced insulin compared to standard diet males, suggesting that mild ketosis of MCT metabolism improved insulin sensitivity in males. In the KD groups, insulin levels were comparably low in both sexes (with no significant sex difference), likely a result of the extreme carbohydrate restriction driving insulin down to baseline in all mice. The overall pattern – males having higher insulin when fed a normal or moderately low-carb diet – aligns with the known greater propensity of males to develop insulin resistance (27). It is known that males generally exhibit lower insulin sensitivity than females, who tend to have more effective insulin-mediated glucose uptake in tissues. These differences are partly rooted in sex hormones: estrogen has well-documented insulin-sensitizing effects, enhancing insulin action in liver, muscle, and adipose tissues (28). Premenopausal females enjoy a degree of protection against insulin resistance, whereas males (or estrogen-deficient females) show higher fasting insulin and greater risk of metabolic syndrome (28). Our findings reflect this paradigm – female mice maintained metabolic homeostasis with lower insulin, while males required higher insulin to control glucose on equivalent diets. The fact that the sex gap in insulin was abolished under KD (when both sexes had very low insulin) suggests that extreme carbohydrate restriction can override baseline physiological differences, essentially “leveling” the insulin requirements. Nevertheless, under more moderate dietary conditions, the female mice had lower insulin, suggesting superior insulin sensitivity or responsiveness. This could translate to practical differences: for instance, a ketogenic or ketone-supplemented diet might allow male subjects to improve their insulin profile (as seen with 15%BHB:MCT males), but female subjects might exhibit little change because their insulin was already low. Overall, the glucose–insulin data imply that female VM/Dk mice are metabolically more insulin-sensitive and maintain tighter glycemic control than males, an observation that mirrors broader sex differences observed in humans (27). These distinctions are important, as they could influence how each sex might benefit from or be challenged by ketone metabolic therapy.

At the level of epigenetic modifications, our study provides evidence that sex also governs the chromatin responses to ketone metabolic therapy, in a tissue-specific manner. In the hippocampus, male mice showed higher levels of global histone lysine acetylation (pan-Kac) and the activating mark H3K4me3 than female mice. This was true overall and was especially pronounced under the KD: KD males had significantly greater pan-acetylation than KD females. H3K4me3, a marker associated with transcriptionally active chromatin, was also elevated in males relative to females in the hippocampus. These epigenetic differences suggest that male hippocampi had a more “open” or transcriptionally permissive chromatin state in response to the ketogenic therapy. Histone acetylation and H3K4 trimethylation both facilitate gene expression by loosening chromatin structure and recruiting transcriptional machinery (29). Thus, the greater pan-Kac and H3K4me3 in males may indicate an increased activation of gene networks (potentially metabolic or neuroprotective genes) in the male brain under ketosis. One possible mechanism for the acetylation difference is the direct effect of ketone bodies on histone-modifying enzymes (30), leading to increased histone acetylation during ketosis or fasting. It is conceivable that in our study, male hippocampal cells were more susceptible to this BHB-mediated hyperacetylation, or achieved higher local BHB levels, than females. If male neurons experienced greater BHB uptake or metabolism, they may have undergone more robust chromatin acetylation changes. Another contributing factor could be baseline epigenetic differences established during brain sexual differentiation. Sex differences in neural histone modifications emerge early in development; for example, even at birth, male rodents have higher levels of acetylated and trimethylated H3 in the cortex/hippocampus compared to females (31). Those innate differences, driven by neonatal testosterone exposure, might persist into adulthood or predispose the sexes to differential epigenomic remodeling. While our study did not pinpoint the exact genes or regions affected, the enrichment of activating histone marks in male hippocampus points to a potential for greater gene expression changes (perhaps in pathways related to ketone utilization, neurotransmission, or neuroprotection). It will be important in future studies to map these modifications to specific hippocampal subregions or even cell phenotypes. The hippocampus comprises metabolically distinct regions (e.g., dentate gyrus, CA1, CA3) and a heterogeneous mix of neurons and glia; dissecting which subpopulation drives the sex difference in acetylation/H3K4me3 could link this molecular dimorphism to functional outcomes (such as cognitive differences or vulnerability to metabolic stress). Overall, the hippocampal epigenetic data reinforce that male and female brains do not respond identically to ketogenic interventions. This again reflects distinct metabolic-pathway engagement between sexes, since histone acetylation and methylation are intimately coupled to cellular metabolic state (30). The male-biased increase in “active chromatin” marks may indicate a greater activation of gene programs in males, but whether this is beneficial (e.g., enhanced expression of neuroprotective factors) or possibly maladaptive is an open question. Regardless, our demonstration of sex-dependent chromatin remodeling in the hippocampus adds a new dimension to understanding how metabolic therapies can differentially affect the male and female brain.

In contrast to the hippocampus, the skeletal muscle (bicep) showed a different pattern of epigenetic response that further underscores tissue-specificity. At baseline (standard diet), female bicep muscle exhibited higher H3K4me3 than male muscle, suggesting females had more active gene chromatin states in muscle under normal conditions. This could correlate with known sex differences in muscle gene expression and physiology – for instance, females often have greater endurance (type-1) fiber type expression and oxidative metabolism in muscle, whereas males have higher muscle mass but different gene activation profiles (32).

Interestingly, this female epigenetic advantage in muscle was abolished by the ketogenic interventions. Neither KD nor 15%BHB:MCT females retained the high H3K4me3 seen in SD females; in fact, 15%BHB:MCT females had significantly lower H3K4me3 than their SD counterparts. Males, on the other hand, showed the opposite trend: 15%BHB:MCT males displayed increased H3K4me3 in muscle compared to SD males. In the KD group, female and male H3K4me3 levels converged, eliminating the sex difference observed on the control diet. These data indicate that ketogenic therapy may induce convergent or even reversing effects on muscle chromatin between sexes. One interpretation is that the muscle of male mice had an epigenetic response to the ketone-supplemented diet that activated certain genes (raising H3K4me3), whereas female muscle did not require or was less able to do so, resulting in a relative reduction in that mark. The net effect was that a sex difference present under a carb-rich SD diet (higher H3K4me3 in females) was attenuated under ketogenic conditions. This could conceivably have functional repercussions. For example, genes involved in muscle growth or oxidative metabolism might be marked by H3K4me3; in SD-fed females those genes could be more active, supporting muscle maintenance. Ketogenic diets might suppress those pathways in females (perhaps due to energy scarcity or hormonal changes), while in males the diets may activate genes (such as stress-responsive or fuel-utilization genes) that were previously lower. Such shifts might influence muscle performance or the progression of cachexia (muscle wasting) in a sex-specific way.

Although speculative, the greater increase of H3K4me3 in male muscle on the 15%BHB:MCT diet could reflect an attempt to counteract muscle stress or to utilize ketone energy, which might be relevant if males are predisposed to muscle catabolism. Conversely, the reduction of H3K4me3 in female muscle by ketosis might indicate a dampening of some anabolic pathways. These hypotheses need investigation at the gene level, but our data clearly show that muscle epigenetic regulation under ketone metabolic therapy is sex-dependent. Importantly, neither global acetylation (pan-Kac) nor the repressive mark H3K9me3 in muscle showed significant changes by sex or diet in our study, implying that the effects were selective to H3K4me3 and specific genomic loci. While our analysis focused on global histone acetylation and methylation patterns, we acknowledge that identifying specific genomic loci enriched for these modifications would provide more mechanistic insight into sex differences in chromatin regulation. Techniques such as chromatin immunoprecipitation (ChIP) targeting acetyl-H3 or H4, followed by high-throughput sequencing (ChIP-seq), could help reveal which gene promoters or regulatory regions are selectively modified in male versus female tissues in response to ketogenic therapy.

Although such experiments were beyond the scope of the current study, we recognize their importance and propose them as a logical next step to extend these findings and define the gene networks underlying the observed sex-dependent epigenetic profiles. We also acknowledge that skeletal muscle is a complex tissue with multiple cell types (myofibers, immune cells, connective tissue) and fiber-type compositions. Future studies using fiber-type specific analysis or single-cell epigenomic methods could identify which muscle cell populations drive the H3K4me3 changes in each sex. That would help tie these epigenetic modifications to muscle function or pathology. Nonetheless, our findings highlight that a given metabolic intervention (ketogenic diet or ketone supplementation) can have divergent epigenetic effects in male versus female muscle. Such divergence further emphasizes that tissue-specific epigenetic responses to diet are modulated by sex (33). The muscle of females and males may activate distinct transcriptional programs when challenged with the same diet, potentially contributing to sex differences observed in outcomes like fatigue, muscle atrophy, or exercise capacity during therapies.

These sex-specific findings in VM/Dk mice highlight important translational implications. Many sex differences observed in preclinical models, particularly in metabolism and drug response, are mirrored in human studies. For example, women often show greater insulin sensitivity and higher ketone levels — trends also seen in female rodents. Clinical cases such as zolpidem, where post-market data prompted sex-specific dosing, underscore the relevance of such differences. While not all preclinical findings translate directly, many reflect conserved mechanisms. Including sex as a biological variable enhances the predictive value of preclinical research and supports development of more personalized therapies.

In summary, this study provides a comprehensive demonstration that sex is a critical determinant of metabolic and epigenetic responses to ketogenic therapy in a preclinical model. Female and male VM/Dk mice differed markedly in key metabolic endpoints – females showed higher ketosis, lower glucose, and lower insulin, whereas males showed blunted ketosis, higher glucose, and higher insulin on equivalent diets. These physiological differences were mirrored by distinct patterns of histone modifications in the hippocampus and skeletal muscle, with males generally exhibiting a greater induction of active chromatin marks in response to the diets (especially in the brain) and females showing unique basal epigenetic profiles that were altered by the interventions (especially in muscle). Collectively, our findings underscore the importance of including both sexes in metabolic research and illustrate that one sex’s response should not be assumed for the other. From a translational perspective, the implications are significant. Ketogenic diets and ketone supplements are being explored as treatments for conditions ranging from epilepsy to cancer. Our data suggest that such metabolic therapies could have sex-dependent efficacy and side-effect profiles. For instance, if female subjects tend to reach higher ketone levels, they might derive greater tumor-suppressive benefits from a ketogenic therapy, but they might also experience metabolic effects (e.g., weight loss or endocrine changes) that differ from males. Males, in contrast, may require more stringent carbohydrate restriction or adjunct measures to achieve the same ketogenic state as females, and they may be more prone to hyperglycemia or insulin resistance if the diet is not well-tailored (26). Considering sex as a biological variable can improve the design of metabolic therapies to maximize benefits and minimize harms for each patient. There is growing recognition that “sex-specific medicine” can enhance personalized treatment strategies (34), and our results strongly support this view in the realm of nutrition and metabolism. Going forward, therapeutic protocols such as ketogenic diets might be optimized by accounting for sex-related metabolic flexibility, hormone milieu, and epigenetic regulation. Developing sex-stratified dietary strategies could involve, for example, adjusting macronutrient ratios, supplementing with exogenous ketones, or monitoring biomarkers (ketones, glucose, lactate, etc.) on different schedules for males vs. females. Recent studies have already revealed that males and females can respond in unexpectedly divergent ways to low-carbohydrate or ketogenic diets (35). Such evidence reinforces that a one-size-fits-all diet therapy is likely suboptimal. Our work contributes to a growing foundation for sex-specific metabolic research, particularly in the context of a cancer-relevant model, and highlights that integrating sex as a fundamental variable will enhance the translational success of metabolic therapies. In conclusion, tailoring ketogenic and other metabolic interventions to the unique biological context of each sex – effectively, a precision nutrition approach – could improve outcomes in diseases like cancer and cachexia. The VM/Dk model data presented here provide a proof-of-concept that sex-specific metabolic and epigenetic phenotypes exist and matter; acknowledging and leveraging these differences will be key in developing the next generation of personalized metabolic therapies.

## Data Availability

The raw data supporting the conclusions of this article will be made available by the authors, without undue reservation.
